# Hypothetical Abductive Reasoning in Dermatology and Dermatopathology

**DOI:** 10.3390/dermatopathology13010003

**Published:** 2025-12-25

**Authors:** Carlo Francesco Tomasini, Lorenzo Magnani

**Affiliations:** 1 Department of Clinical-Surgical, Diagnostic and Pediatric Sciences, University of Pavia, 27100 Pavia, Italy; 2 Dermatology Clinic, Fondazione IRCCS Policlinico San Matteo, 27100 Pavia, Italy; 3 Department of Humanities, Philosophy Section and Computational Philosophy Laboratory, University of Pavia, 27100 Pavia, Italy; lorenzo.magnani@unipv.it

**Keywords:** abductive reasoning, clinicopathologic correlation, Select-and-Test model, visual and manipulative abduction, pattern recognition, PALMs, algorithmic method, Ackerman, artificial intelligence

## Abstract

Abductive reasoning, or *abduction*, is a key process in scientific discovery and medical diagnosis. In everyday dermatology and dermatopathology, however, it functions as the practical engine behind differential diagnosis, clinicopathologic correlation, and disciplined pattern recognition. In this paper, we retain the epistemological foundation of abduction but translate it into usable steps for clinicians and dermatopathologists. We distinguish abduction from deduction and induction; separate *creative abduction* (which generates new concepts) from *selective abduction* (daily diagnostic choice); and show how both operate within a simple Select-and-Test (ST) Model: select a hypothesis, deduce what else should be true, test against data, and then update. We then reinterpret Ackerman’s algorithmic method of pattern analysis as an operationalization of the ST-Model. Through a couple of concise case vignettes, we illustrate visual and manipulative abduction, nonmonotonic updates, and the role of artifacts (dermoscopy, DIF, stains) as so-called epistemic mediators. Finally, we map contemporary AI tools to selective abduction and propose practical guardrails for fairness, transparency, and accountability. The result is a pragmatic framework that preserves philosophical depth while addressing the daily needs of dermatologists and dermatopathologists in the clinic and at the microscope.

## Simple Summary

Abductive reasoning (abduction) is the core inferential process in scientific discovery, medical diagnosis, and everyday dermatology and dermatopathology. It drives differential diagnosis, clinicopathologic correlation, and pattern recognition. Unlike deduction, which applies rules to reach certain conclusions, or induction, which generalizes from repeated observations, abduction generates new ideas that must later be tested empirically. Two main forms of abduction in reasoning are selective abduction (choosing from known hypotheses) and creative abduction (generating new concepts). Both are framed in a simple Select-and-Test (ST) Model: select a hypothesis, deduce testable predictions, test against data, update beliefs (nonmonotonically). In this paper, Ackerman’s classic pattern-analysis algorithm is reinterpreted as an operational ST-Model. A case vignette demonstrates visual/manipulative abduction and the role of epistemic mediators. Contemporary AI tools are mapped to selective abduction, with proposed guardrails for fairness, transparency, and accountability. The result is a pragmatic, epistemologically grounded framework tailored to daily dermatology and dermatopathology practice at the bedside and microscope.

## 1. Abductive Reasoning in Dermatology and Dermatopathology

Abduction—from the ancient Greek *ἀπαγωγή*, meaning “to lead away”—is often presented as a strictly philosophical topic. In fact, it is a key process in scientific discovery and medical diagnosis. Introduced by the 19th-century American philosopher Charles Sanders Peirce, it refers to a syllogism in which the major premise is certain, while the minor premise is only probable [1]. Unlike deduction, which applies rules to reach certain conclusions, or induction, which generalizes from repeated observations, abduction generates new ideas that must later be tested empirically.

Human thought has three fundamental forms of reasoning and logical inference—the mental process of moving from what we believe to be true (information) to what else we believe to be true—used to draw conclusions from data and observations: deduction, induction, and abduction. Deduction starts from a general premise (rule) to arrive at a specific conclusion, guaranteeing its truth if the premises are true. Induction generalizes from specific observations to a general rule or law, but its conclusions are probable, not certain. Abduction is a process of inference that produces the “best explanation” for a set of observations by formulating a probable hypothesis (Table 1).

Examples:

- **Deduction**: Rule → Case → Result
  “All beans in this bag are white → These beans are from this bag → These beans are white.”
- **Induction**: Case → Result → Rule
  “These beans are from this bag → They are white → All beans in this bag are white.”
- **Abduction**: Result → Rule → Case
  “These beans are white → All beans in this bag are white → They come from this bag.”

Abduction expands knowledge by suggesting plausible explanations. For instance, before, we only knew the beans were white; now, we can also suppose they come from this bag. While powerful, abduction is also prone to error since it deals with uncertainty and creative hypothesis generation. If we see broken glass on the floor, we might explain it by postulating that wind blew it over. This is not a deductive consequence of the glass being broken—a cat may just as well have been responsible.

Thus, theoretical abduction is the process of inferring facts, laws, or hypotheses that render observations plausible and that explain—or even discover—new phenomena. It is the reasoning process in which explanatory hypotheses are formed and evaluated. Peirce emphasized that abduction is both diagnostic and inventive: it enables physicians and scientists to interpret observations and propose new concepts, forming the foundation of much medical reasoning [2,3].

### 1.1. Why Abduction Matters in Dermatology and Dermatopathology

Diagnostic reasoning never begins with certainty. The dermatologist or dermatopathologist starts with partial, noisy observations—an eruption’s distribution, a lesion’s color variegation, or a biopsy’s silhouette at scanning magnification—and proposes an explanation. That proposal is an abductive hypothesis. Deduction then derives what else should be true if the hypothesis holds (for example, expected dermoscopic or histologic clues).

What Peirce calls *induction*—here understood as confrontation with data, not as generalization (as explained above)—then checks those predictions against evidence from microscopy, special stains, direct immunofluorescence, serology, imaging, or even therapeutic challenge. The cycle is fast, iterative, and—crucially—open to revision.

Abduction differs from the other inferential modes. Deduction preserves truth: if the premises are true, the conclusion must also be true. Induction generalizes from repeated observations to a rule and gains strength only with further data. Abduction, by contrast, ventures a plausible explanation before certainty is available. It is fallible by design, which is precisely why it is useful in medicine. Dermatology features many-to-one mappings from manifestation to cause: scales, for example, may arise from psoriasis, eczema, tinea, or pityriasis rubra pilaris. Abduction is the disciplined way we move from “what it looks like” to “what it most likely is,” while keeping alternatives open until evidence decides.

### 1.2. Creative Versus Selective Abduction

The term *abduction* has two primary meanings:

1. abduction as the generation of “plausible” hypotheses (either *selective* or *creative*), and
2. abduction as inference “to the best explanation,” which also incorporates the evaluation of hypotheses [2,3].

**Figure 1:**
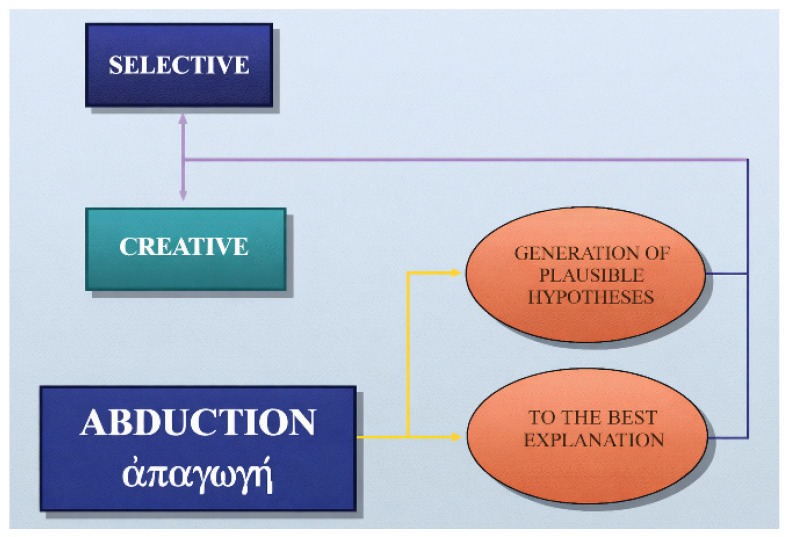
Creative and selective abduction.

Creative abduction expands medical knowledge and opens routes for research and therapy by coining new nosological entities or reconceptualizing old ones. Selective abduction, in contrast, is the daily work of diagnosis: choosing the most plausible label from a known list. Creative abduction is rare but transformative; selective abduction is constant and life-saving (Figure 1).

### 1.3. A Syllogistic Snapshot (Why Abduction Is Risky but Necessary)

In dermatopathology, the triad of deduction, induction, and abduction can be expressed in the following syllogistic forms:

- **Deduction**: “If psoriasis is present, parakeratosis and thinning of the granular layer will be present; psoriasis is present; therefore, those findings are present.”
- **Induction**: “We have repeatedly observed parakeratosis with psoriasis; therefore, psoriasis tends to produce parakeratosis.”
- **Abduction**: “Parakeratosis is present; therefore, psoriasis might be the cause.”

The abductive move is invalid in classical logic because other diseases also show parakeratosis. Yet it is essential for hypothesis generation. Diagnosis proceeds by risking a conjecture and then testing it against data.

### 1.4. Base Rates, Pathognomony, and the Cost of Being Wrong

Two refinements help clinicians and dermatopathologists use abduction wisely.

**Figure 2:**
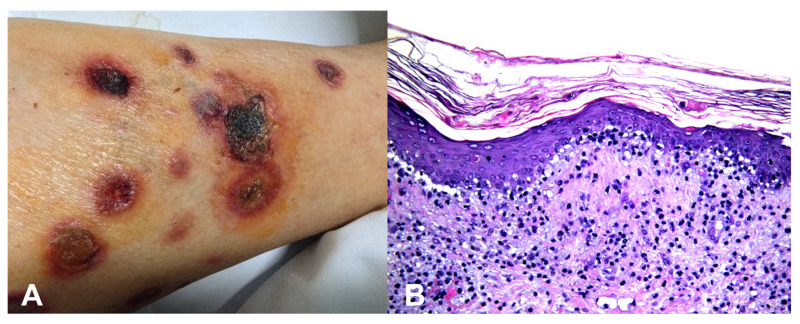
(**A**) Primary cutaneous aggressive epidermotropic CD8+ cytotoxic T-cell lymphoma presenting with large, targetoid, partly ulcerated plaques resembling atypical erythema multiforme. (**B**) Vacuolar and lichenoid interface dermatitis-like changes. The condition was initially misinterpreted as atypical erythema multiforme, leading to diagnostic delay with a catastrophic outcome.

**Base rates**: When two hypotheses explain the finding equally well, the more common disease in the given setting (season, geography, clinic type) is preferred. However, always keep a rare “zebra” on the list if missing it would be catastrophic. For example, an atypical erythema multiforme-like eruption with a tendency to ulcerate may represent a primary cutaneous aggressive epidermotropic CD8+ cytotoxic T-cell lymphoma (Figure 2).

**Figure 3:**
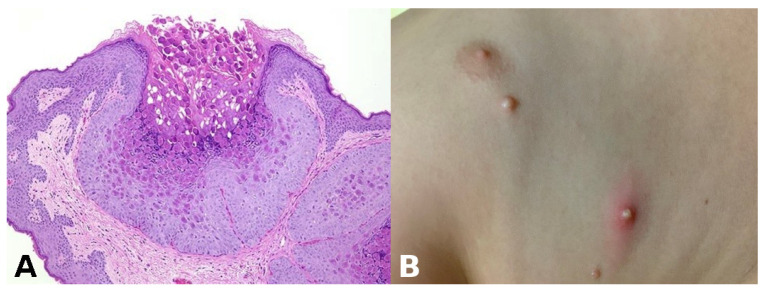
(**A**) Henderson–Paterson (molluscum) bodies: large intracytoplasmic eosinophilic inclusion bodies within keratinocytes. (**B**) White to flesh-colored, dome-shaped, pearly, umbilicated papules of molluscum contagiosum.

**Pathognomonic evidence:** This refers to a particular sign, symptom, or finding whose presence is sufficient for a diagnosis, leaving no room for doubt or alternative interpretations. For example, the presence of Henderson–Paterson bodies within a dilated infundibulum is pathognomonic for molluscum contagiosum (Figure 3).

A pathognomonic sign is **bidirectionally tight** (A ↔ B). In such cases, abduction collapses toward certainty, and the ST-Model (explained below) can proceed rapidly. Much more often, however, evidence is only partially discriminative, and the safe course is to document alternative interpretations and plan tests that most efficiently separate them.

## 2. The Select-and-Test (ST) Model, Made Practical

**Figure 4:**
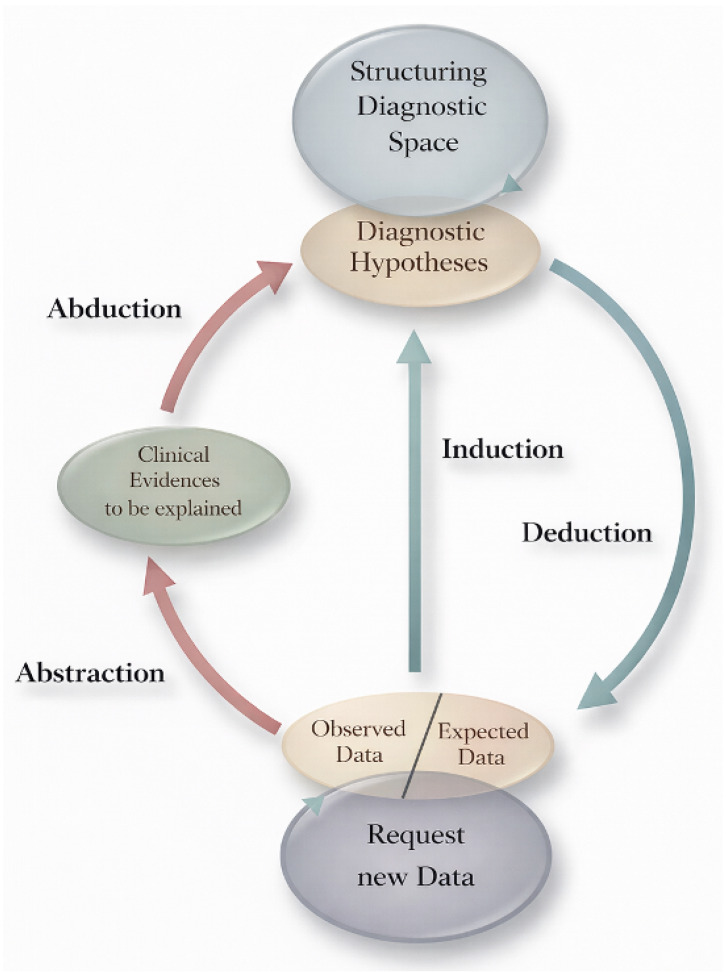
Select-and-Test (ST) Model. Modified from Ramoni et al. [2].

The ST-Model is an epistemological framework first developed by Ramoni et al. in 1992 [2] and later elaborated by Magnani [3] (Figure 4). It compresses medical reasoning into three iterative moves that loop until stability:

1. **Select**—Propose abductively one or more hypotheses that can explain the observations. In inflammatory dermatopathology, this often begins with pattern recognition: perivascular, spongiotic, lichenoid (interface), psoriasiform, vesiculobullous, nodular/diffuse, vasculitic, or panniculitic.
2. **Deduce**—From each hypothesis, infer additional findings that should be present. For psoriasis, these include regular elongation of rete ridges, thinning of suprapapillary plates, neutrophils in the stratum corneum (Munro microabscesses), and a thinned granular layer; clinically, there are well-demarcated erythematous plaques on extensor surfaces with silvery scale.
3. **Test**—Correlate the predictions with patient-specific data: histology at higher power, special stains, culture or PCR, dermoscopy, direct immunofluorescence (DIF), serology, imaging, or therapeutic trials. Keep a simple “scoreboard”: which hypothesis gains support, and which loses?

**Figure 5:**
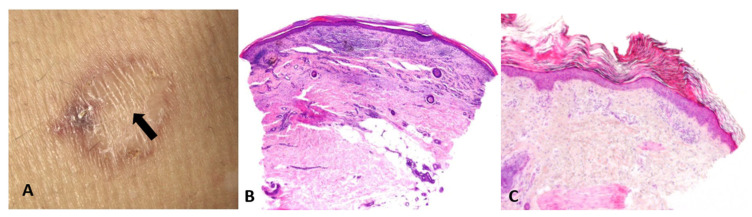
(**A**) Biopsy from the regressing center of a porokeratosis lesion (arrow). (**B**) Nondiagnostic pathology, showing lichenoid infiltrate, fibrosis, and angiogenesis. (**C**) Biopsy from the elevated border of a porokeratosis lesion, revealing the characteristic cornoid lamella—a column of parakeratotic cells that represents the hallmark of the condition.

Two properties of the ST-Model are especially important for safety:

- **Nonmonotonicity**: Adding new premises can invalidate previous conclusions. A lesion that appeared consistent with pyoderma gangrenosum yesterday may prove to be leishmaniasis today once a travel history is elicited or a new stain is performed, as illustrated in the Case Vignette (Section 6).
- **Artifacts and actions matter**: Biopsy site selection, technique (punch vs. shave vs. excision), depth, and handling are not mere logistics; they are integral to reasoning itself—manipulative abduction. Actions generate the very data required for the next abductive step. For example, a common sampling error in daily dermatologic practice is taking a biopsy from the central area of porokeratosis (Figure 5).

The ST-Model has been effectively applied in pedagogy through the diagnosis software developed at Massey University for teaching plant disease diagnosis [4]. Over a decade of use in plant pathology courses, surveys consistently showed strong student engagement, improved understanding of diagnostic reasoning, and preference for interactive learning over traditional lectures. Qualitative feedback emphasized realistic scenarios and valuable feedback mechanisms, though empirical generalization is limited by small sample sizes.

Complementary research by Vimla L. Patel on medical cognition reinforces these findings [5]. Her studies on problem-based learning (PBL) demonstrate superior diagnostic accuracy, faster hypothesis formation, and better knowledge retention compared to lecture-based curricula. For instance, PBL students showed a 25–30% increase in diagnostic efficiency and 40% improvement in reasoning accuracy with structured training [6]. Across decades of research, Patel’s work supports the pedagogical benefits of hypothesis-driven, metacognitive approaches—aligning closely with the principles of the ST-Model. Together, these empirical results highlight the model’s potential to enhance critical reasoning and expertise development in diagnostic education. Building on this evidence, we propose to apply the ST-Model-based pedagogical approach to dermatopathology education for residents in dermatopathology.

## 3. Visual and Manipulative Abduction: How Seeing and Doing Think for Us

Dermatology is a medical discipline primarily based on visual perception—the brain’s active process of interpreting and organizing light signals from the eyes to construct a meaningful understanding of the environment [5]. Perception is much more complex than a mere topographical representation of sensory input. It involves cognitive functions that process shapes, colors, spatial relationships, and movements, enabling recognition, problem-solving, and interaction with the world.

Closely related to visual perception is **visual abduction**, an active cognitive process that leverages perceptual power to detect patterns, relationships, or anomalies that trigger the generation of explanatory hypotheses [7]. It plays an important cognitive role in both everyday reasoning and scientific work and is especially crucial in dermatological and dermatopathological cognition.

Visual abduction transforms perceived images into hypotheses: a reticular pigment network suggests a melanocytic lesion; arborizing vessels suggest basal cell carcinoma; vesicles on an erythematous base evoke contact dermatitis. Dermatopathologists follow the same process at the microscope: a perivascular superficial and deep infiltrate of lymphocytes and eosinophils suggest insect bite reaction, but may also indicate drug hypersensitivity reaction, eosinophilic folliculitis, lymphomatoid papulosis, or Wells’ syndrome—all prompt explanatory guesses that must then be tested.

However, visual abduction differs among individuals and is influenced by the quality of visual perception. Accurate diagnosis of skin biopsies requires not only specialized knowledge but also the pathologist’s ability to perceive and interpret images, identify critical regions within the tissue, and appropriately classify visual features into candidate histological diagnoses.

**Figure 6:**
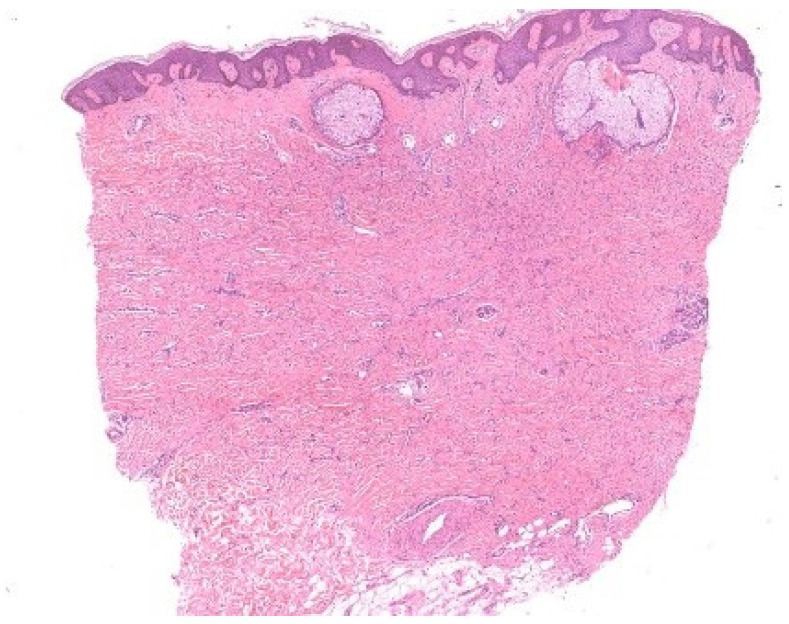
Figure–ground segregation. In this dermatofibroma with sebaceous gland induction, the round, simple structures of the sebaceous glands contend with the ill-defined proliferation of fibroblasts in the dermis, which lacks a continuous contour.

Böer demonstrated that unconscious perceptual rules, particularly figure–ground segregation, govern what is consciously noticed under the microscope [8]. This process allows us to isolate figures against a background but can also mislead: the fibroblast proliferation of a dermatofibroma is readily detected by an expert, while a novice may be distracted by incidental sebaceous induction and overlook the true lesion (Figure 6). Likewise, subconscious mechanisms of visual perception may cause interstitial infiltrates—especially when sparse—to receive less attention than epidermal, perivascular, or perifollicular infiltrates [9].

These observations resonate with the notion of semi-encapsulated perception argued by Raftopoulos [10] and with Magnani’s eco-cognitive model of abduction [3]. Early visual processing remains cognitively independent, but later stages integrate knowledge. Novices attend to simple figures, while experts exploit tacit knowledge to prioritize ground features. Still, expertise does not abolish error: dermatofibrosarcoma protuberans can be mistaken for dermatofibroma when unconscious biases prompt premature closure. Böer’s studies underscore that unconscious processes are constitutive of abductive reasoning but are also sources of diagnostic pitfalls. Training that enforces systematic scrutiny of “ground” details can counteract these biases and refine abductive precision.

## 4. Enhancing Novices’ Abductive Proficiency in Dermatopathological Inflammatory Diseases

This logic extends to pedagogy. Rimoin et al. showed that Perceptual and Adaptive Learning Modules (PALMs) accelerate pattern recognition in pre-clerkship medical students [11]. The system presents a series of images in short classification tests, requiring students to categorize them based on visual patterns. This process reflects abductive reasoning: students encounter a new image (observation), draw on prior knowledge of pathological characteristics (background knowledge), and hypothesize the correct category (e.g., inflammation) based on perceived patterns.

Moreover, the PALM system, as described by Krasne et al. for teaching introductory dermatopathology, has an adaptive nature [12]. It adjusts the sequence and distribution of images based on students’ accuracy and response times, supporting abductive reasoning by directing attention to relevant features and reinforcing pattern recognition over analytical reasoning. By dynamically adjusting image presentation and providing immediate feedback, PALMs cultivate the rapid hypothesis generation characteristic of expert visual abduction. In the web-based module developed by Krasne, diagnostic skills not only improved significantly but were retained after one year, suggesting that structured exposure can compress the long arc of clinical apprenticeship [12].

Therefore, PALMs exemplify how abductive cognition—normally honed tacitly over years—can be trained explicitly, supporting the transition from analytical feature recognition to intuitive, hypothesis-driven perception. This iterative process mirrors Magnani’s view of abduction as a dynamic, hypothesis-driven cycle that improves with experience [3].

**Manipulative abduction** is “thinking through doing.” Dermoscopy, reflectance confocal microscopy, tape stripping for PCR, DIF sampling, and simply choosing a second biopsy from the advancing edge of an annular plaque are epistemic actions. They extend the mind with artifacts, producing information unattainable by passive viewing alone. When planned as deductions from hypotheses—“If it is lupus, DIF at the lesional margin should show granular IgG/C3 along the DEJ”—they discipline the process: the test is chosen for a reason, and its result is interpreted against a specific prediction.

Purely visual reasoning falters when disease stage, prior therapy, scratching, or infection reshapes morphology. Manipulative steps—holding topical steroids before biopsy, sampling the edge of a fresh blister for H&E and perilesional skin for DIF, ordering PAS in the presence of parakeratosis with neutrophils—restore the original signal. Even the requisition form matters: a photograph attached to a biopsy is not decoration but a data-bearing artifact that legitimately shifts abductive probabilities.

## 5. Ackerman’s Algorithmic Method Inside the ST-Model

**Figure 7:**
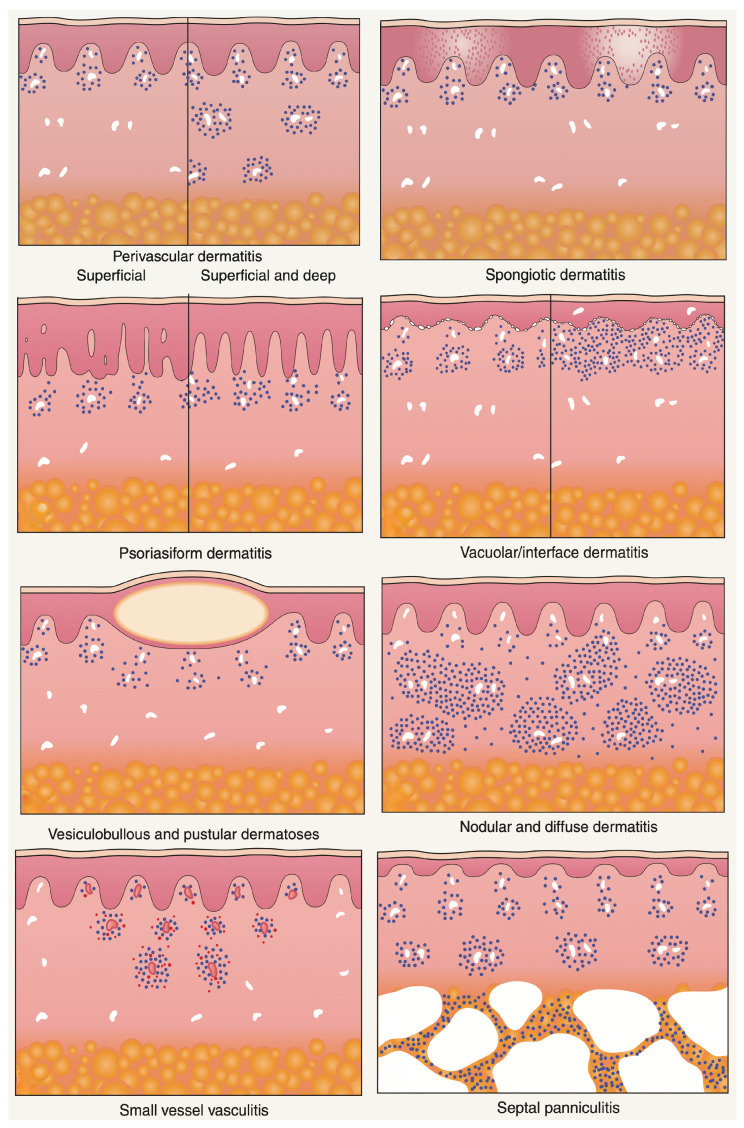
Major histopathologic patterns of cutaneous inflammation. Adapted from A. B. Ackerman, *Histologic Diagnosis of Inflammatory Skin Diseases: A Method by Pattern Analysis*, Lea & Febiger, Philadelphia, 1978 [13].

Albert Bernard Ackerman championed an algorithmic approach to pattern analysis of inflammatory skin diseases that harmonizes with the ST-Model [2,3]. His method begins with low-power architecture—the “pattern”—and proceeds to high-power cytology and distribution [13]. It helps the dermatopathologist identify diagnostic shortcuts and differentiate disorders with similar presentations. Correlation with a clinical setting is obligatory throughout. The point is not to trap the diagnostician in a rigid tree but to stage abductive choice, deductive prediction, and inductive testing in an orderly way (Figure 7).

### 5.1. Major Inflammatory Patterns (with Abductive Triggers)

- **Superficial perivascular and superficial and deep perivascular dermatitis**—Looser infiltrates that require clinical context and often special stains to avoid overcalling. Abductive triggers: not scaling erythematous–edematous patches/plaques.
- **Spongiotic dermatitis**—Intercellular edema with exocytosis; clinically eczematous plaques with scale crust. Abductive triggers: pruritus, oozing vesicles; dermoscopy with yellow crusts.
- **Psoriasiform dermatitis**—Regular acanthosis and elongated rete ridges. Abductive triggers: sharply demarcated erythematosus scaling patches and plaques.
- **Interface dermatitis**—Basal vacuolar changes at the dermal–epidermal junction with or without band-like lymphocytes obscuring the DEJ, Civatte bodies. Abductive triggers: itchy, red, dry, or blistering maculopapular rashes, drug exposure, autoimmunity, photosensitivity.
- **Vesiculobullous dermatitis**—Intraepidermal or subepidermal splits. Abductive triggers: flaccid vs. tense blisters, mucosal involvement; DIF strategy differs accordingly.
- **Nodular and diffuse dermatitis**—Dense inflammatory aggregates, granulomas, pseudolymphomas. Abductive triggers: nodules, draining sinuses, foreign body, infectious agents.
- **Vasculitis**—Vessel wall damage with fibrinoid necrosis and leukocytoclasia. Abductive triggers: palpable purpura, systemic symptoms, drug/viral exposure.
- **Panniculitis**—Inflammation involving the subcutaneous fat, with lobular and/or septal distribution. Abductive triggers: tender, deep-seated nodules and plaques on the lower extremities.

Ackerman’s insight was pedagogic: start broad, then narrow. Each pattern invites a shortlist of abductive hypotheses. Each hypothesis dictates deductions (what else to look for) and tests (DIF, PAS, AFB, Fite, Alcian blue, elastin stains, PCR). In other words, his algorithm is a scaffold for the ST-Model.

### 5.2. Example Worked Through the Cycle: Psoriasis

- **Select (abduction)**—Low-power psoriasiform pattern suggests psoriasis among the candidates.
- **Deduce**—Expect thinning of the suprapapillary plates, loss of the granular layer, neutrophils in the stratum corneum (Munro microabscesses) or in spongiotic foci (Kogoj pustules), and tortuous dilated papillary capillaries.
- **Test**—Confirm with high power; correlate with clinical distribution (extensors, scalp), nail changes, and response to therapy. If spongiosis dominates or parakeratosis is patchy with fungal hyphae on PAS, pivot toward eczema or tinea, respectively.

### 5.3. Non-Specific Findings as Vehicles of Abduction in the Algorithmic Method

Another route to coming to specific diagnosis in inflammatory dermatopathology is emphasized by Ackerman in chapter seven of his book *Histologic Diagnosis of Inflammatory Skin Diseases* (3rd ed.)—i.e., by recognizing “non-specific” findings as a vehicle to diagnosis [14]. Indeed, diagnostic reasoning in dermatopathology may begin not from a broad low-power pattern, but from the recognition of individual microscopic clues—sometimes subtle and apparently “non-specific.” These findings, though insufficient for a definitive diagnosis, have cognitive value: they act as triggers for the abductive process that underlies the algorithmic method.

**Figure 8:**
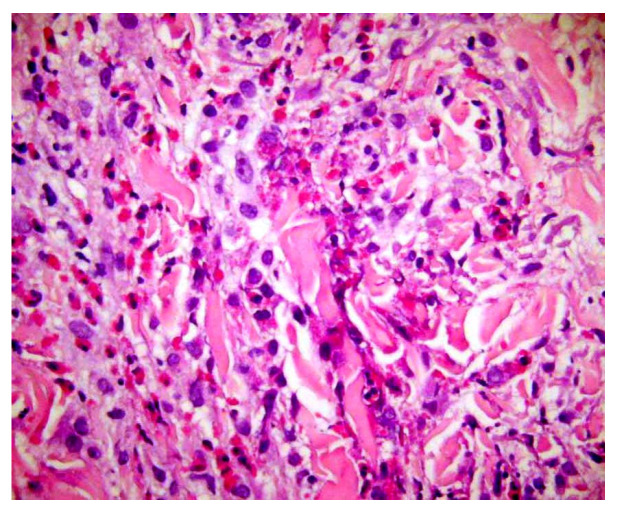
Flame figures: degenerated dermal collagen bundles coated with eosinophilic granules, which give them a bright red color resembling a flame. Though characteristically described in Wells’ syndrome, they are not pathognomonic of this disease, but can be encountered in any condition with numerous eosinophils, including bullous pemphigoid, insect bite reaction, scabies, eosinophilic folliculitis, drug reactions, mycotic infections.

Examples include **abundant mucin in the reticular dermis**, **acantholytic dyskeratotic cells within a cleft**, **flame figures**, **sarcoidal granulomas**, or **eosinophilic abscesses**. None of these features is pathognomonic: each may occur in several unrelated entities. Yet, when observed, they stimulate the diagnostician to *select* a set of plausible hypotheses, *deduce* the additional features that should accompany each hypothesis, and *test* them through further histologic levels, special stains, or clinicopathologic correlation (Figure 8).

Within this Select-and-Test (ST) framework, such indeterminate clues serve as **vehicles for abductive reasoning**. They generate **weak abductions**—tentative, exploratory explanations that define a hypothesis space rather than a conclusion. For instance, identifying eosinophilic abscesses may raise competing hypotheses such as Wells’ syndrome, eosinophilic folliculitis, or a drug reaction. Abduction organizes this uncertainty: the observer asks, “What else should I see if this is Wells’ syndrome? Is there flame-figure formation, collagen degeneration, or a peripheral eosinophilia?” Deductive prediction and empirical testing then refine or overturn the initial guess.

Conversely, when a **specific or pathognomonic feature** is present—such as Henderson–Paterson bodies or cornoid lamellae—abduction collapses toward **strong abduction**, where explanation and verification converge almost immediately. In most inflammatory dermatoses, however, Ackerman’s genius was to convert the commonplace and non-specific finding into a disciplined reasoning tool: rather than discarding ambiguous findings, he incorporated them into an ordered inferential cycle.

In this way, the algorithmic method and the ST-Model coincide philosophically. Both treat uncertainty as the essential condition of diagnosis, not its failure. The non-specific finding becomes the **epistemic entry point**—the spark that launches abduction and transforms mere description into understanding. Each clue, however modest, thus participates in constructing the best possible explanation for the lesion at hand, until the pattern stabilizes into a coherent diagnostic hypothesis.

### 5.4. Why the Algorithm Is Not Enough (and Why It Is Still Essential)

Pattern analysis reduces noise but cannot exhaust reality. Stage of disease, treatment, scratching, and secondary infection alter histology. Terminology proliferates (“pathobabel”), and novices may mistake labels for explanations. Ackerman’s remedy—structured observation plus correlation—does not eliminate uncertainty; it makes uncertainty productive by forcing explicit abductive updates.

## 6. Case Vignette That Teaches the Cycle

### Pyoderma Gangrenosum That Was Not

**Figure 9:**
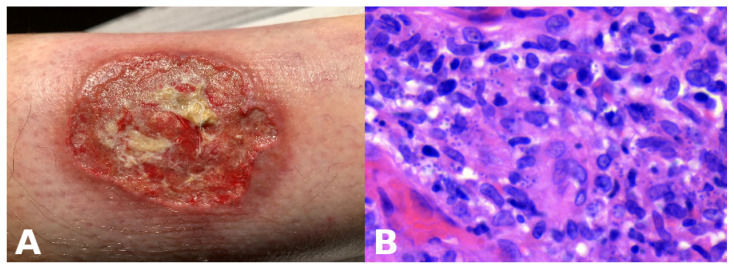
Ulcerative cutaneous leishmaniasis misinterpreted as pyoderma gangrenosum. (**A**) A large cutaneous ulcer with a bleeding, fibrinoid bed and an irregular, violaceous, undermined border. (**B**) High magnification demonstrating *Leishmania* amastigotes within macrophages.

- **Presentation:** Rapidly enlarging ulcer with undermined violaceous borders and severe pain (Figure 9A).
- **Abductive step:** Pyoderma gangrenosum (PG) is high on the list.
- **Deduction:** Expect sterile neutrophilic dermal infiltrates; history of IBD or arthritis; paradoxical worsening with surgical debridement; response to steroids.
- **Test:** Biopsy shows neutrophilic and granulomatous infiltrate (Figure 9B), but treatment fails. Travel history reveals exposure risk. Re-evaluation of sections at high power and Giemsa stain identifies intracellular parasites; PCR confirms *Leishmania*.
- **Update:** New abductive hypothesis—cutaneous leishmaniasis—supersedes PG; itraconazole leads to cure.
- **Lesson:** Nonmonotonicity is not diagnostic failure; it is diagnostic hygiene. Therapy response is itself a test within the ST-Model.

## 7. Making Abduction Safer: Teamwork, Language, and Specimens

### 7.1. Clinicopathologic Communication as Part of Inference

A requisition that lists “rule out psoriasis vs. eczema” without distribution, evolution, drugs, or prior treatment starves the abductive cycle. Provide duration, sites, systemic symptoms, therapies tried, travel and exposure history, comorbidities, and clinical photographs. Dermatopathologists should, in turn, signal uncertainty explicitly (“favored diagnosis,” “cannot exclude”) and state what data would best test the leading hypotheses (repeat biopsy, DIF, special stains, culture) [15].

### 7.2. Guarding Against “Pathobabel” and Cognitive Traps

- **Anchoring**—First impressions drive subsequent steps. *Counter*: write at least two rival hypotheses.
- **Availability**—Recent memorable cases bias selection. *Counter*: use checklists keyed to patterns.
- **Representativeness**—Overweighting “classic” features. *Counter*: ask what would make the hypothesis unlikely.
- **Language inflation**—Proliferating synonyms and borderline entities obscure decision making. *Counter*: tie terms to observable criteria and management consequences.

### 7.3. Biopsy as Epistemic Engineering

- **Site**—Sample the active edge or youngest lesion; avoid fully treated areas.
- **Technique**—Punch deep enough for adnexa or panniculus when relevant; excise pigmented lesions where architecture matters.
- **Handling**—Separate specimens for H&E, DIF, culture/PCR when needed.
- **Documentation**—Mark orientation and attach photographs. Each step widens eco-cognitive openness and reduces the chance of abductive dead ends.

## 8. What AI Adds—And What It Does Not

Modern AI tools in dermatology and dermatopathology largely perform selective abduction at scale. Trained on many labeled images or spectra, a model maps a new input to a probability over known categories. When the top probability crosses a threshold, the system proposes a diagnosis or risk label (“suggestive of melanoma/BCC/SCC”). In spectroscopy-based devices, the data come from subsurface optical signatures rather than images, but the abductive move is the same: select the most plausible explanation from a stored repertoire and offer it to the clinician [16,17].

AI already assists with triage and decision support, yet three limits parallel human abductive fallibility:

- **Bias and coverage**—If training sets underrepresent skin of color, pediatric lesions, or rare variants, the model’s hypotheses will be skewed. The fix is not only more data but better governance: curated diversity, transparent reporting, and routine external validation.
- **Opacity**—Heatmaps or saliency maps offer post hoc rationales but may not match histopathologic ground truth. Bridging methods—weakly supervised localization, prototype-based reasoning—bring AI explanations closer to reusable clinicopathologic predicates (e.g., “irregular pigment network,” “arborizing vessels”).
- **Nonmonotonic updates**—Like human reasoning, a model’s outputs should change when new inputs appear (a dermoscopic view, a biopsy result, a time series of images). Systems that cannot ingest sequential evidence risk freezing abduction at step one. Integrations that allow “evidence updates” align AI with the ST-Model and with real practice.

A practical template is to treat AI outputs as a second reader. For a pigmented lesion, the clinician’s abductive shortlist (melanoma vs. nevus vs. SK) is generated first. An AI score then enters as one more piece of evidence on the ST-Model scoreboard. When the AI disagrees with the clinician, a rule of engagement might be to escalate uncertainty (biopsy rather than observe) or to seek a second human opinion. For dermatopathology images, experimental systems that highlight regions driving the classification are easier to audit and accept. Workflows that capture pre-test probability (the clinician’s abductive prior) and post-test probability (after the AI output) encourage calibrated decisions rather than yes/no jumps.

Ethically, clinicians remain accountable. AI outputs should be treated as hypotheses, not verdicts, and explicitly placed on the abductive scoreboard alongside clinical and histologic data. Documenting why an AI suggestion was accepted or rejected builds institutional memory and patient safety. Regulatory clearance, where present, signals a specific indication, not blanket accuracy; indications should be treated as boundaries for safe abduction, not permission to turn off judgment.

## 9. Pearls: A Pocket ST-Model for Daily Use

1. Name the pattern (clinical and histologic).
2. List at least two hypotheses that fit.
3. Write two predictions per hypothesis you could check today.
4. Order one test or action that would most decisively separate them (biopsy placement counts).
5. Record the result and update—in writing.
6. If therapy is started, treat the response as a test, and be ready to reverse course.

This simple scaffold externalizes abduction, trains residents, and curbs bias without slowing the clinic or the sign-out room.

## 10. Limitations and Scope of This Review

This article focuses on the abductive structure common to everyday dermatoses and neoplasms and on Ackerman’s pattern analysis because these topics are teachable and actionable. We do not attempt a full taxonomy of inflammatory patterns or an encyclopedic review of AI literature. Disease names evolve, criteria are contested, and therapeutic landscapes shift. The ST-Model accommodates such change precisely because it regards conclusions as provisional. Readers should adapt the checklists and vignettes here to local prevalence, resources, and expertise.

## 11. Conclusions

Abduction is not a luxury of philosophy; it is the practical logic of dermatologic diagnosis. The ST-Model renders that logic explicit: select, deduce, test—then repeat without embarrassment when new data arrive. Ackerman’s algorithmic method operationalizes the cycle for histopathology by anchoring abduction in reproducible patterns while insisting on clinicopathologic correlation. Visual and manipulative abduction remind us that seeing and doing are themselves forms of thinking, and that well-engineered artifacts—from dermatoscopes to DIF—are part of cognition, not mere accessories. AI systems now join the workflow as large-scale engines of selective abduction; used as second readers with clear guardrails, they can widen eco-cognitive openness without displacing human responsibility.

Dermatopathologists can learn that diagnosis is not just spotting facts—it is making educated guesses (abduction) to explain tricky skin findings. Unlike straight-line deduction (if A, then B) or pattern-matching induction (this looks like that before), abduction starts with “what if?” to build the best story from clues like a biopsy’s shape or a rash’s look. It splits into creative (new ideas for rare cases) and selective (picking from common suspects daily). The big win? It is flexible—new info can flip your guess, avoiding stuck thinking. In daily practice, use the simple Select-and-Test (ST) model to stay sharp: Select a top guess (e.g., psoriasis from scaly plaques). Deduce what else fits (thinned skin layer, nail pits). Test it fast (check stains, patient history, or dermoscopy). Update if wrong—like swapping pyoderma for leishmaniasis after a travel clue. Blend in Ackerman’s patterns: scan at low power for big shapes (spongy, blistery), then zoom in. Add “manipulative” tricks: biopsy the edge, not center; attach photos to reports. Follow these pearls: name the pattern, list two or more options, predict checks, run one key test, note results, and track therapy tweaks. This curbs biases, trains teams, and welcomes AI as a “second eye” for probabilities. The result? Fewer misses, faster sign-outs, and teachable humility—turning “maybe” into safe care.

## Figures and Tables

**Table 1:** Types of human reasoning.

	Deduction	Induction	Abduction
Type of inference	Necessary inference	Probable inference	Most plausible explanation
Characteristics	Draws specific conclusions with certainty	Generalizes from observations; uncertain	Best guess based on facts and logical reasoning; uncertain
Reasoning style	Top-down reasoning	Bottom-up reasoning	Generates new ideas

## Data Availability

There is no new data were created. Data sharing is not applicable.
